# Valorisation of Protein from Underutilised Harbour Crab (*Liocarcinus depurator*) as an Egg Yolk Replacer in Mayonnaise-like Sauce Production

**DOI:** 10.3390/foods14234084

**Published:** 2025-11-28

**Authors:** Marta María Calvo, Ana Isabel Román-Cabrera, Oscar Martínez-Alvarez

**Affiliations:** Institute of Food Science, Technology and Nutrition (ICTAN, CSIC), 6th Jose Antonio Novais St, 28040 Madrid, Spain; mmcalvo@ictan.csic.es (M.M.C.);

**Keywords:** *Liocarcinus depurator*, discards, harbour crab, egg-yolk substitute, functional protein, seafood upgrading, mayonnaise, emulsifier, valorisation

## Abstract

The present study aimed to valorise the harbour crab (*Liocarcinus depurator*), a common bycatch species, by evaluating its protein as an egg yolk substitute in a mayonnaise-type sauce. The harbour crab was firstly characterised for its physicochemical and elemental composition (including amino acid profile, macrominerals, trace, and heavy metals). Protein was extracted by precipitation at the isoelectric point (with or without a previous delipidation step), and the foaming and emulsifying properties of the resulting protein extracts were evaluated. The method that did not involve prior delipidation resulted in a higher protein extraction yield. Furthermore, this method resulted in a protein extract with superior emulsifying activity. This protein extract was used as an egg-yolk replacer in the preparation of a mayonnaise-type sauce. Sensory evaluation revealed that the sauce formulated with crab protein was comparable to the egg-based control in terms of overall flavour and acceptability, but only with the addition of curry to mask the seafood taste. The results of this study demonstrate that crab meat protein, when extracted without previous lipid removal and combined with curry, can be used as a viable egg yolk substitute in mayonnaise-type sauces.

## 1. Introduction

Discards represent a significant global issue within the fishing industry, defined as the unintended bycatch captured alongside target species. A considerable portion of these discards consist of species with low commercial value or sizes below the regulated minimum, including several species of crustaceans such as crabs. According to the Food and Agriculture Organization (FAO) [1], between 1950 and 2022, catches of edible marine species encompassed 1234 crustacean species, of which 336 were distinct crab species. Gilman et al. [2] estimated that global marine capture fisheries discard 9.1 million tonnes of seafood per year, representing 10.8% of the average catch between 2010 and 2014. Notably, certain discarded species, such as hermit crabs and other crustaceans, have negligible commercial value. However, other species, including the mud crab (Scylla species), edible crab (*Cancer pagurus*), and Chinese mitten crab (*Eriocheir sinensis*), are highly valued worldwide for their high protein content and distinctive flavour. These discards present environmental and economic challenges, necessitating valorisation strategies for sustainable seafood production systems.

The common harbour crab (*Liocarcinus depurator*) is a soft-shell species commonly found in the North Sea, Atlantic Ocean, Mediterranean Sea, and Black Sea. It is often captured as bycatch and subsequently discarded due to its low commercial value. Harbour crabs exhibit a pronounced depth-related distribution, with the highest recorded densities found on the continental shelf between 51 and 100 m (approximately 985 individuals per square kilometre) [3]. A substantial decline in abundance is observed at the upper slope (201–300 m), with an average of approximately 130 individuals per square kilometre. Their abundance becomes negligible below 500 m, where the species accounts for less than 10% of the catch. This spatial distribution pattern, when considered alongside the fact that commercial trawling operates across a broad depth range, suggests that incidental capture exerts limited pressure on the overall population. Species aggregation in specific depth strata, coupled with its absence from deeper habitats, reduces the likelihood of sustained depletion. Consequently, bycatch mortality is less impactful than that of taxa with wider or more uniform distributions. Although the common harbour crab possesses culinary potential, it has traditionally been less commercially valuable than other crab species. Its morphological characteristics have been documented [4,5,6]. These include a small size (20.4–32.3 mm), weight (4.1–8.3 g), and a low muscle mass ratio [6], a characteristic that often results in its being overlooked in commercial fisheries. In certain Mediterranean countries, it is commonly available at low prices, primarily for use in culinary broths.

However, the relatively low consumption of harbour crabs necessitates the development of novel applications that can valorise these discards and mitigate the environmental impact associated with bycatch. García-López et al. [7] reported the richness of crabs in astaxanthin and omega-3 fatty acids, highlighting their potential as a source of these valuable compounds for aquaculture feed. Furthermore, and similar to other shellfish [8], the harbour crab is a valuable source of protein. Crab proteins, like other marine proteins, can be extracted using a range of methodologies, including chemical approaches such as acid or alkaline precipitation, physical techniques like ultrasound or microwave-assisted extraction [9], supercritical fluids, pressurised liquid, pulsed electric fields, ultrasound, membrane technology, team explosion [10], and isoelectric solubilisation/precipitation [11]. Nonetheless, careful processing is essential to preserve protein functionality and limit undesirable characteristics such as fishy odours [12]. Many of these protein extraction strategies offer significant potential for application in the food industry, particularly where marine proteins can serve as substitutes for traditional ingredients due to their techno-functional properties. This is especially pertinent in the context of mayonnaise, where egg yolk is traditionally employed as an emulsifier alongside vegetable oil, acidic components, flavouring agents (such as salt and mustard), stabilisers, and texture enhancers. Replacing egg yolk with a marine-derived emulsifying protein presents several distinct advantages. Economically, the supply of eggs has been periodically destabilised by avian influenza outbreaks, leading to substantial increases in global egg prices. Additionally, foods containing eggs are susceptible to microbial contamination, primarily by *Salmonella* sp., and spoilage [13,14]. Consequently, food manufacturers and researchers are exploring alternatives from both plant (e.g., potato starch) and animal sources (e.g., whey protein, casein, meat and seafood proteins) [15] to reduce production costs while simultaneously extending product shelf life [16,17]. From a nutritional perspective, this substitution would enable the production of mayonnaise devoid of cholesterol and with a more favourable lipid profile by eliminating the saturated and specific fats present in egg yolk [14]. Furthermore, it provides an alternative for consumers with egg allergies, thereby enhancing market accessibility, although it is important to note that a small segment of the population may have allergies to myofibrillar proteins from seafood [18]. Moreover, the marine protein may function as an effective emulsifier, ensuring the structural stability of the mayonnaise and maintaining a smooth and uniform texture without the need of synthetic stabilisers. A previous study has demonstrated that fish roe protein concentrates with high emulsifying capacity can successfully replace egg yolk in mayonnaise, resulting in a product with high stability, desirable viscosity, and small droplet size [19]. Similarly, Rajasekaran et al. [20] partially replaced egg yolk by myofibrillar protein from shrimp, obtaining a mayonnaise with good functional and sensory properties. Proteins from other marine sources, including harbour crabs, may also exhibit strong emulsifying properties and be used as egg yolk replacers in mayonnaise and other food products.

This study aimed to evaluate the effectiveness of two methods for extracting protein from the harbour crab, as well as the extraction yield, techno-functional properties, and the potential of the extracted protein to replace egg yolk in a mayonnaise-type sauce.

## 2. Materials and Methods

### 2.1. Raw Material

Harbour crabs (*Liocarcinus depurator*) were captured by trawling in autumn off the coast of Castellón (Spain) in the Mediterranean Sea. The crabs were purchased on the day of capture and transported to Madrid at low temperatures (4 °C). Upon arrival, they were washed, frozen at −40 °C in a cold tunnel and stored at −80 °C until use.

### 2.2. Determination of the Proximate Composition

The crabs (1 kg total, average weight 10 ± 3 g) were thawed overnight at 4 °C and homogenised using a Thermomix TM31 (Vorwerk, Wuppertal, Germany) food processor until a uniform mass was obtained (9 sec. at speed 6). No water or buffer was added during this step. A portion of the homogenate was lyophilised using a Beta 1-8 LSCbasic freeze-dryer (Christ, Osterode am Harz, Germany). The condenser temperature was set to −50 °C, and the vacuum pressure was stabilised at 0.05–0.20 mbar. The freeze-drying process was maintained for a total of 96 h.

Moisture content of the crab homogenate was determined in triplicate according to AOAC Official Method 950.46 [21]. The results were expressed as grams of water/100 g of sample.

The ash content of the crab homogenate was determined in triplicate following the AOAC Official Method 942.05 [21]. The results were expressed as grams of ash/100 g of wet sample.

The protein content of the crab homogenate and the dried fractions was determined in triplicate using the Dumas method, measuring the nitrogen content according to the AOAC Official Method 990.03 [21]. A Leco TruMac Nitrogen analyser (Leco Corp., St Joseph, MI, USA), calibrated with EDTA, was used. A nitrogen-to-protein conversion factor of 6.25 was used. The results were expressed as grams of protein/100 g of wet sample (homogenate) or dried sample (protein fraction).

Sugar content of the crab homogenate was determined using the anthrone method, as described by Loewus [22]. The sample was previously lyophilised. It was mixed with distilled water at a ratio of 1:10 (*w*/*v*). Then 3 mL of 96% sulfuric acid containing 0.2% anthrone was added. The mixture was heated in a water bath at 95 °C for 15 min. After cooling to room temperature, the absorbance was measured at 610 nm. Glucose was used as a standard, processed in the same manner as the sample. The results were expressed as grams of sugar/100 g of wet sample. Sugar content was determined in triplicate.

Lipid content in the crab homogenate was determined in triplicate according to Bligh and Dyer [23]. The homogenate was previously freeze-dried, and further mixed with a solution of methanol, chloroform, and water in a volumetric ratio of 2/1/0.8 (*v*/*v*/*v*) and a weight ratio of 1/9 (*w*/*v*) for 1 h at room temperature. The liquid phase was then separated from the solid residue using a sieve, and the liquid phases were separated using a decantation funnel to obtain the lipid fraction in the chloroform phase. This procedure was performed in triplicate on the solid residue from the previous extraction. Chloroform was then evaporated using a rotary evaporator (R-300, BÜCHI, Flawil, Switzerland). The fat content was calculated by difference in weight and expressed as grams of fat/100 g of wet sample.

### 2.3. Determination of Amino Acid Composition

The homogenate was freeze-dried, dissolved in ultrapure water, filtered, and hydrolysed under vacuum at 110 °C for 24 h in the presence of HCl 6N with 0.1% phenol and norleucine as an internal standard. The hydrolysate was then freeze-dried, dissolved in the application buffer and analysed for amino acid composition using a Biochrom 30 amino acid analyser (Biochrom Ltd., Cambridge, UK) equipped with an LKB Ultrapack resin column (Pharmacia LKB Biotechnology, Inc., Pascataway, NJ, USA). The results were expressed as number of residues per 1000 residues.

### 2.4. Evaluation of the Content of Simple Sugars

The content of simple sugars (glucose, galactose, fructose, sucrose, and fucose) in the crab homogenate was analysed using ion chromatography. The homogenate was freeze-dried and mixed with ultrapure water at a ratio of 1/10 (*w*/*v*) and vacuum-filtered. The equipment used for this study consisted of a Bioscan module 817 IC (Metrohm, Herisau, Switzerland) with an amperometric pulse detector (945 Professional Detector Vario), a pump (IC Pump 812), a coupled degasser (IC-837), and an injector (889 IC Sample Center). The control software employed was Metrodata IC Net 2.3 and MagiIC Net 2.3. The column used was a MNetrosep Carb 2 column (250 × 4 mm × 5 μm particle size), and the mobile phase consisted of NaOH 300 mM with NaOAc 1 mM, at a flow rate of 0.5 mL/min for 50 min. A PAD detector was employed. Quantification was achieved by analysing the area of the peaks and comparing then with simultaneously eluted. The results, averaged over three determinations, were expressed as mg/kg of dried sample.

### 2.5. Evaluation of the Content in Minerals and Heavy Metals

The content of macrominerals (Na, K, Ca, Mg, P, and S), trace elements (Fe, Cu, Mn, and Zn) and heavy metals (Pb, Cd, As, and Hg) in crabs was determined using atomic absorption spectroscopy (ContrAA700, Analytik Jena GmbH, Jena, Germany). Prior to analysis, the crabs were homogenised, freeze-dried, and digested with nitric acid and hydrogen peroxide in a microwave oven. The results were expressed as g/kg of the sample for sodium (Na), potassium (K), calcium (Ca), and magnesium (Mg) and as mg/kg of the sample for phosphorus (P), sulphur (S), iron (Fe), copper (Cu), manganese (Mn), zinc (Zn), chromium (Cr), lead (Pb), cadmium (Cd), arsenic (As), and mercury (Hg). The determinations were performed in triplicate.

### 2.6. Optimisation of the Protein Extraction Process

#### 2.6.1. Protein Extraction

Proteins were isolated by isoelectric point precipitation before or after lipid extraction. In the extraction procedure prior to lipid extraction (method A), the homogenised freeze-dried sample was mixed with distilled water in a 1/4 ratio (*w*/*v*), and the pH was adjusted to 12 using NaOH 5M. The selection of pH 12 was based on a previous study investigating protein solubility at different basic pH values (see the following section for details). Following a 20 h stirring period at a low temperature of 4 °C, the mixture was centrifuged at 9200× *g* for 30 min at 4 °C (Sorvall Evolution, Thermo Fisher Scientific, Waltham, MA, USA). The protein extraction process was repeated two additional times on the precipitate obtained after centrifugation. All protein extracts were mixed and filtered under vacuum, and the pH was adjusted to 4.5 using 6 M HCl. After 20 h of stirring at a low temperature (4 °C), the extract was centrifuged at 9200× *g* for 30 min at 4 °C. The protein precipitate obtained was resuspended in sodium phosphate buffer (0.1 M, pH 7.5) and stirred at 4 °C. The soluble protein was then freeze-dried.

Protein extraction according to method B was performed using the lipid-free shell and muscle fragments. The protein was precipitated at its isoelectric point, as described in method A, and this process was repeated three times. The precipitated protein was resuspended in sodium phosphate buffer (0.1 M, pH 7.5) and freeze-dried until use. The amount of protein extracted by each method was calculated based on the nitrogen content using a conversion factor of 6.25, as indicated in Section 2.2. The protein extraction yield was calculated from this value and expressed as a percentage relative to the initial protein content of the dried raw material.

#### 2.6.2. Determination of the Isoelectric Point (pI) and pH of Maximum Solubility

The isoelectric point of the protein extracted using method A was determined as follows: The protein solution was first diluted in distilled water (1/100, *v*/*v*), and the pH was adjusted to different values from 2 to 12 using 0.2 M HCl. Protein solubility was determined using a Zetasizer Nano Z590 (Malvern Instruments, Ltd., Malvern, UK) with the ‘Zeta Potential’ function. The data were corrected for the Brix levels of the homogenised crabs. All measurements were performed in triplicate. The Zeta potential (mV) was then determined, defined as the electrostatic potential at the boundary layer of dispersed particles. Values close to 0 mV indicate that the pH is near the isoelectric point of the protein. Conversely, high Zeta potential values (either positive or negative) reflect strong electrostatic repulsion, leading to higher colloidal stability and enhanced protein solubility.

### 2.7. Evaluation of the Techno-Functional Properties of Proteins

The techno-functional properties of the freeze-dried protein obtained by each of the two methods (A and B) were evaluated in terms of foaming and emulsifying capacity, using commercial albumin as a control.

#### 2.7.1. Foaming Capacity and Stability

The foam expansion (FE) and stability (FS) were evaluated at different protein concentrations (1% and 2%), following the methodology described by Ketnawa et al. [24]. The freeze-dried proteins and albumin were dissolved in 10 mL of distilled water and homogenised for one minute at 14,000 rpm using an ULTRA-TURRAX Tube Drive homogeniser (IKA Werke GmbH & Co., Staufen, Germany) to form a foam in a 50 mL cylinder with a diameter of 4.5 cm. The height reached by the foam was measured at various time intervals (0, 1, 5, 10, 20, 30, and 60 min).

The foam expansion (FE) was determined using the following formula:

$$FE\left(\%\right)=\frac{\left(V_{T}-V_{0}\right)}{V_{0}}\times 100$$

where V_T_ corresponds to the volume after homogenisation and V_0_ to the volume before stirring.

The foam stability (FS) was calculated using the following formula:

$$FS\left(\%\right)=\frac{\left(V_{T}\times 100\right)}{V_{0min}}$$

where V_T_ corresponds to the volume after 60 min and V_0min_ to the volume at 0 min after homogenisation. The trial was conducted in triplicate at a controlled temperature (24 °C), and the results were expressed as the mean and standard deviation.

#### 2.7.2. Emulsifying Capacity and Stability

The Emulsifying Activity Index (EAI) and Emulsifying Stability Index (ESI) were determined according to the protocol outlined by Ketnawa et al. [24], with minor modifications. The analysis encompassed two distinct protein concentrations (0.5% and 1.5%). The freeze-dried proteins and albumin were dissolved in 3 mL of distilled water, along with 1 mL of sunflower oil, and homogenised for 1 min at a rate of 14,000 rpm to form an emulsion. Subsequently, aliquots (100 μL) were taken at 0 and 10 min and diluted in 10 mL of 0.3% sodium dodecyl sulphate (SDS). The resulting samples were then subjected to spectrophotometric analysis (Shimadzu UV-1601, Tokyo, Japan) at a wavelength of 500 nm. The emulsifying activity index was determined using the following formula:

$$EAI\left(\frac{m^{2}}{g}\right)=\frac{\left(2\times 2.303\times DF\times A\right)}{\left(C\times \theta \times 10000\times \emptyset \right)}$$

where DF is the dilution factor (133.3), A is the measured value of the optical density at 500 nm, C is the protein concentration (g/mL), ∅ is the ratio between the volume of the dispersed phase (oil) and the total volume of the emulsion (0.25), and θ is the path length (0.01 m).

The absorbance of the emulsion measured at 0 and 10 min was used to calculate the ESI using the following formula:

$$ESI\left(min\right)=\frac{A_{0}}{∆A}\times ∆t$$

where ∆A = A_0_ − A_10_, and ∆t corresponds to 10 min.

The trials were conducted in triplicate, and the results were expressed as the mean and standard deviation.

### 2.8. Elaboration of the Product

Three mayonnaise-type sauces were formulated as detailed in Table 1. The control sauce was prepared using whole eggs (size M, average weight 55 ± 2 g), whereas the other two sauces incorporated the protein extract with the best emulsifying properties (extract obtained using the method A), with one variant including curry. The control sauce was prepared by gradually adding oil to the egg in a tall beaker, while homogenising the mixture using a blender. For the other two sauces, the protein extract was first homogenised with the egg white using an ULTRA-TURRAX Tube Drive homogeniser at 1000 rpm for 20 s. The mixture was then transferred into a tall beaker, where oil was gradually added and the emulsion was formed using a blender, following the same procedure as for the control sauce. In the curry-flavoured sauce, curry powder was added after the emulsion had been properly formed. The nutritional composition of the ingredients used to prepare the mayonnaise sauces (Table 2) was calculated using the information provided by the U.S. Department of Agriculture [25].

### 2.9. Sensory Analysis

The attributes and acceptability of the products were evaluated using descriptive analytical sensory analysis. In this study, each sample was assigned a code, and a tasting sheet was used to record the main attributes to be assessed for each product using a numerical scale from 1 (strongly dislike) to 9 (strongly like). Sensory analysis was conducted in a sensory analysis room by 12 pre-trained panellists. The selection of these panellists was made to ensure maximum representation of the target population segments in terms of age (21–61 years) and sex (7 women, 5 men). The attributes evaluated were emulsion homogeneity, consistency, and appearance (visual phase); odour and curry aroma (odour phase), greasy mouthfeel and taste (mouth phase); overall flavour; and overall acceptability.

### 2.10. Statistical Analysis

The emulsifying and foaming activities of the protein extracts and control were compared using one-way ANOVA. A post hoc test (Tukey HSD) was applied to determine which pairs differed from each other. Statistical analyses were performed using SPSS 27 software (IBM Corporation, Armonk, NY, USA). The significance level was set at *p* ≤ 0.05. All measurements were performed in triplicate to ensure accuracy.

## 3. Results and Discussion

### 3.1. Chemical Composition Analysis of Harbour Crabs

#### 3.1.1. Proximate Composition

The moisture content of the crab homogenate was 71.9 ± 1.2%, which is slightly lower than the value reported for the muscle of brown crab, *Cancer pagurus* (74.6–77.8%) [26] and for lump of mud crab, *Scylla paramamosain* (78.7–84.4%) [27]. The findings of this study fall within the ranges reported in the literature for different parts of crab species, such as the mud crab, where the moisture content ranged from 79.7% in muscle to 46.5% in gonads [28]. In a separate study, Zotti et al. [29] documented moisture content values ranging from 74.2% to 80.9% for eight distinct crab species.

Generally, crab species exhibit higher protein levels compared to *Liocarcinus depurator*, which has a protein content of 9.2 ± 0.9%. The observed low protein content could be attributed to the significant presence of shell material in the homogenate. It is important to note that protein content can vary within the same species depending on the physiological state of the animal. For instance, post-molted crabs (soft-shell) typically have lower protein content than pre-molted crabs (hard-shell) [27]. Additionally, variations in protein content among crab species are influenced by factors such as season, body size, sex, reproductive maturity, food availability, and geographical distribution [30]. Comparable low protein contents have been documented in soft-shell mud crabs (*Scylla oceania*, 8.33%) [31]. In other crab species, protein values have been observed to range from 14.3% to 33.4% [29,32,33]. Wang et al. [28] reported protein levels of 10.6–33.4% in different edible tissues of female mud crabs (*Scylla paramamosain*). Benjakul and Suthipan [27] reported a protein content of 14.3–15.6% in hard-shell mud crabs (lump meat and claws), whereas these values were lower in soft-shell crabs (12.9% in lump meat and 3% in claws). Czerniejewski et al. [32] reported a protein content between 13.6 and 24.2% in Chinese mitten crab, depending on the edible part analysed. Furthermore, Gökoõlu and Yerlikaya [33] documented protein levels between 12.3% and 22.6% in various crab species.

The ash content (14.6 ± 1.2%) was considerably high and much higher than that described for other crab species, which ranged between 1.55% and 2.55% [29]. Conversely, other studies have documented lower ash contents in the brown crab (*Cancer pagurus*) (1.6–6.6%) [34] and the Chinese mitten crab (1.4–1.9%) [35]. The high ash content observed in the harbour crab may be explained by the use of the whole animal for analysis, since both the shell and legs exoskeleton contain substantial amounts of minerals, which in turn contribute to the higher ash values. Additionally, the absorption of seawater may also partially account for the high mineral content.

The lipid content (1.9 ± 0.6%) was similar to that observed in other crab species, although this content varied considerably (0.7% to 20.5%) depending on the part analysed (muscle, hepatopancreas, or gonads), with the highest content being found in the hepatopancreas and gonads [28].

About the carbohydrate content, it was low (0.4% ± 0.0%), showing values similar to those reported for different crab species (0.17.3.4%) [30].

#### 3.1.2. Amino Acid Composition

The amino acid composition of the whole harbour crab revealed the prevalence of glycine, followed by aspartic acid and asparagine, glutamic acid and glutamine, and alanine (Table 3). This profile is consistent with previous findings in other crab species, such as the Chinese mitten crab [36], the mud crab (*Scylla tranquebarica*) [37] and the blue crab (*Callinectes sapidus*) [38]. The high levels of glutamic acid/glutamine and aspartic acid/asparagine suggest the presence of muscle proteins from breast and claw meat in the homogenate [38]. The abundant glycine content strongly indicates the presence of collagen in crustacean muscle. This hypothesis is reinforced by the significant amounts of proline and lysine (and their hydroxylated forms), which are characteristic of collagen and other structural proteins, as well as by the notable contribution of alanine [39,40].

The essential amino acid content in whole harbour crab was approximately 30%. The most abundant essential amino acid was lysine (63 residues/1000, 6.3%), followed by leucine (61/1000, 6.1%). The amino acid profile may vary depending on the physiological state of the animal. Sudakhar et al. [30] reported a higher abundance of essential amino acids in hard shell crabs, of methionine in newly moulted soft-shell crabs (*Portunus sanguinolentus*), and of leucine in the same species at the pre-moult (hard-shell) stage. The high proportion of branched-chain amino acids (leucine, isoleucine, and valine) may be related to enhanced protein and energy metabolism of this small crab species, given its need for rapid movements to escape predators and capture prey [41,42]. Leucine promotes protein synthesis in muscle [41], and is required for the formation of leucine-enkephalin, a neurotransmitter that induces the secretion of the moulting hyperglycaemic hormone in crabs [43]. Valine promotes amino acid metabolism, supports muscle growth, and enhances energy metabolism by driving glycogen consumption [42]. 

The total amount of hydrophobic amino acids was approximately 35%. A hydrophobic proportion of approximately 30–40% is typical of marine invertebrate muscle proteins, and the value observed in harbour crab falls within this expected range. This proportion aligns with the composition of crustacean myofibrillar proteins, where hydrophobic residues may contribute to structural stability and facilitate the formation of tightly packed protein domains [44].

#### 3.1.3. Carbohydrate Composition

The carbohydrate fraction in crab tissues is predominantly composed of chitin, with minor quantities of glycosaminoglycans covalently linked to proteins, and glycogen [45,46]. Although simple sugars can be present in crabs, their concentration is minimal. In harbour crab, ion chromatography detected low amounts of glucose (232 ppm) and fucose (179 ppm). Additionally, the presence of glucosamine/galactosamine (234 ppm) was observed, supporting the occurrence of glycosaminoglycans; however, these amino sugars could not be quantified or individually identified due to identical elution times. Other monosaccharides, such as galactose, fructose, mannose, arabinose, xylose, and rhamnose, as well as disaccharides like trehalose and maltose, were not detected. Conversely, a peak corresponding to an unidentified sugar was observed at a retention time close to that of other disaccharide standards. This disaccharide may consist of glucose and/or fucose units. Another unidentified component accounted for the major peak and could correspond to a trisaccharide according to its retention time, similar to standards such as raffinose, stachyose, or verbascose. In the context of trisaccharides previously described in crustaceans, this peak could potentially correspond to oligosaccharide chains formed by fucose and glucose, as supported by the detection of these monosaccharides in the sample.

#### 3.1.4. Mineral Content

The mineral profile of crabs can vary significantly depending on factors such as species, habitat, and diet. Even within the same species, mineral composition may fluctuate according to developmental stage and environmental conditions, as crabs are able to absorb minerals through their gills. In the analysed harbour crabs, the average mineral profile revealed significant concentrations of various essential elements (Table 4).

The Ca content was remarkably high (11.1 g/kg), which can be attributed to the composition of the exoskeleton, abundant in calcium carbonate [47]. Conversely, Elegbede and Fashina-Bombata [48] documented higher Ca concentrations (ranging from 15.8 to 38.1 g/kg) in the carapace of *Callinectes pallidus* and *Cardisoma armatum*, and between 3.8 and 18.9 mg/kg in the edible portions. Besides calcium, other essential macrominerals, such as Na and P, were found at high concentrations. The Mg content was also high owing to its high concentration in the exoskeleton of crustaceans. In other crab species, such as the Chinese mitten crab, the predominant minerals identified included Ca, Mg, Na and K [49].

With regard to trace elements, the high Fe content was remarkable, with values exceeding 12 mg/kg, as well as that of Zn, at around 5 mg/kg, and Cu, around 2.5 mg/kg. The abundance of Zn, Fe, Cu, and P has been previously reported in Chinese mitten crab [49]. Zn, Fe and Cu could mainly accumulate in muscle tissue, gill and hepatopancreas [50], as observed in the blue crab. They are absorbed from the surrounding water during the moulting process and incorporated into the formation of the new exoskeleton. For this reason, its concentration is higher in hard shell crabs compared with soft shell crabs [27].

Crabs are macrobenthic organisms that can accumulate heavy metals through their feeding habits and prolonged contact with contaminated sediments, where these elements tend to concentrate in aquatic ecosystems. Consequently, the levels of heavy metals in crabs vary depending on environmental conditions, including the characteristics of the surrounding habitat and, in some cases, temperature-related factors that influence metal bioavailability and uptake [50]. Some heavy metals (Cd, Cr, Pb, As, Hg) are mainly concentrated in hepatopancreas [50], as observed in the blue crabs. In harbour crab, the concentrations of Cd and Hg remained below the maximum limit of 0.5 mg/kg set by the European Union [51] and the US Environmental Protection Agency [52]. This is significant for human health, as excessive consumption of crabs with elevated metal concentrations poses risks. However, the Pb concentration in crustaceans exceeded the legal limit of 0.50 mg/kg of fresh weight [51,52]. It is important to note that these values were obtained from the whole animal, whereas the legal limits only apply to the meat content of the appendages.

Furthermore, a high total As content was observed, which aligns with the findings for other crab species, where arsenic is often present due to environmental factors [53]. However, in crustaceans and other aquatic animals, this mineral is usually found mostly in organic forms (mainly as arsenobetaine), which are generally non-toxic to humans. Inorganic arsenic (As_III_ and As_V_) is considered the most toxic form, and its concentrations are usually low and do not pose a risk to human health, as indicated by the EFSA and different authors [54,55,56].

### 3.2. Protein Extraction and Solubility at Different pHs 

Method A yielded a higher protein recovery (74.2 ± 8.7%) than method B (51.9 ± 10.9%), indicating that protein extraction is more efficient when no prior lipid removal is performed. This finding may be explained by two factors. First, the lipid-extraction step in Method B can co-extract protein bound to lipids and carotenoids, thereby reducing the amount of protein available for the subsequent extraction step. Second, the chloroform/methanol solvent system used in Method B may alter protein conformation, decreasing its solubility during aqueous extraction and further lowering yield. It is important to note that each method has practical trade-offs. The alkaline extraction used in Method A precludes subsequent lipid recovery due to saponification, whereas Method B allows lipid extraction but at the cost of reduced protein yield.

The amino acid profiles of the proteins extracted using methods A and B (Table 3) exhibited slight disparities from those of the raw material. Both extraction methods led to a pronounced reduction in glycine (from 142 mg/100 g in the raw material to 94 and 73 mg/100 g, respectively), proline, and hydroxyproline. This pattern is characteristic of the limited recovery of collagen-derived proteins, which is expected considering the low solubility and structural rigidity of native collagen [40]. Conversely, the glutamic acid/glutamine content (Glx) increased in both extracts, especially in method B (173 mg/100 g), along with a marked increase in branched-chain amino acids (valine, isoleucine, and leucine). This enrichment suggests that the extraction process (particularly method B) favoured the recovery of myofibrillar proteins, such as actin and myosin, which contain higher levels of these amino acids. Regarding the proportion of essential amino acids (36–37%), it increased in both extracts compared with the raw material (30%, Table 3).

The total amount of hydrophobic amino acids in the extracted protein (36–37%) was higher than that in the raw material (33%), consistent with a greater extraction of myofibrillar proteins, which reflects an improvement in the nutritional quality of the extracted proteins.

**Figure 1:**
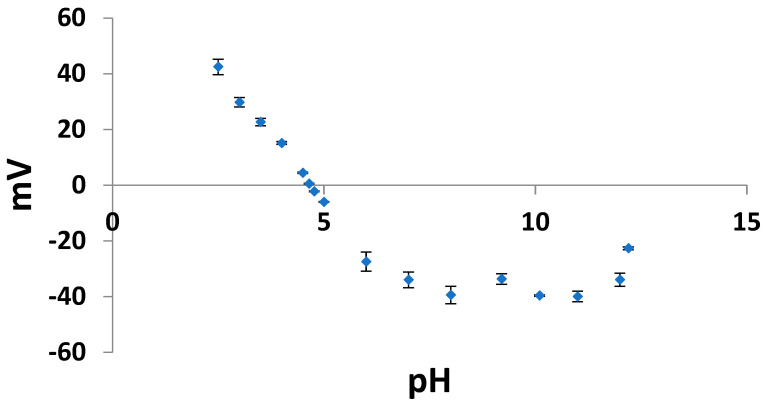
Z potential values of the protein extract in the pH range of 2–12, expressed in millivolts (mV).

About the isoelectric point (pI) of the crab protein, it was 4.65 (Figure 1), corresponding to the pH at which the Z potential reached zero millivolts (mV). This value is consistent with the established pI range of 4.5–5.5 for major myofibrillar proteins, including actin, myosin, troponin, and tropomyosin [57], but notably lower than the pI documented for collagen of different marine species (approximately 6–7) [58].

Myofibrillar proteins constitute a significant portion of muscle tissue, and their pI values significantly influence the overall pI of the extracted protein. Notably, crab protein exhibited high solubility under both acidic (pH 2.5) and alkaline (pH 8, 10, and 11) conditions. While proteins typically exhibit minimum solubility at their pI, the high solubility observed at pH 2.5 and under alkaline conditions suggests that other factors must be considered. At pH 2.5, the protein has a net positive charge due to the protonation of acidic amino acid residues, and the electrostatic repulsion between these positively charged protein molecules may contribute to increased solubility, thereby preventing aggregation and precipitation. Conversely, under alkaline conditions (pH 8, 10, and 11), the protein has a net negative charge due to the deprotonation of the basic amino acid residues.

### 3.3. Assessment of Techno-Functional Properties

#### 3.3.1. Foaming Expansion and Foaming Stability

**Figure 2:**
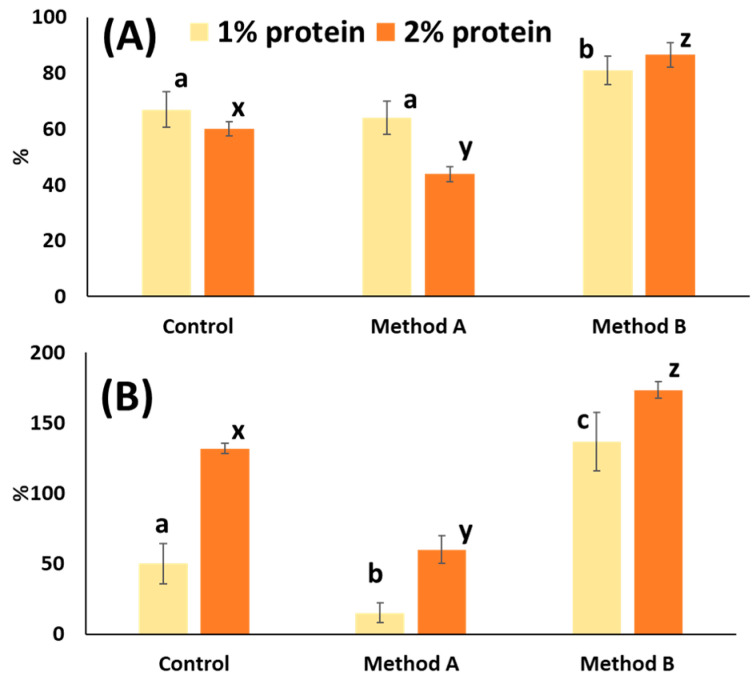
(**A**) Foaming stability of protein extracts obtained using methods A and B at two different concentrations (1% and 2%), compared with commercial albumin (control); (**B**) Foaming expansion capacity of protein extracts obtained using methods A and B at two different concentrations (1% and 2%), compared with commercial albumin (control). Different letters for the same protein concentration (a, b, c or x, y, z) indicate significant differences among samples (*p* < 0.05).

The ability of the proteins extracted using methods A and B to incorporate air at different concentrations was assessed, and the results are shown in Figure 2A. Statistical analysis indicated that, at a concentration of 1%, the foaming stability of the protein obtained using method B was significantly higher than that of the others, reaching 81% after 60 min, whereas the protein obtained using method A and the control showed similar values.

The foaming stability of the protein extracted using method B was once again significantly the highest at a concentration of 2% (Figure 2A), achieving 86.6%, which surpassed the values observed at 1% protein concentration. In addition to its superior stability, the protein from method B also exhibited the greatest foaming expansion capacity at both 1% and 2% (Figure 2B), outperforming the control and the protein extracted using method A. These findings suggest that crab protein obtained via method B is capable of forming stable foams by effectively reducing surface tension and creating a viscoelastic film around air bubbles. This enhanced foam stability may be attributed to differences in protein size and flexibility. Smaller proteins and peptides diffuse rapidly and adsorb quickly at the air–water interface, promoting foam formation but generating thin, low-viscosity films that collapse easily [59,60]. In contrast, larger proteins tend to unfold at the interface, forming cohesive and viscoelastic layers that enhance foam stability. Therefore, it is plausible that the proteins extracted using method A contained a higher proportion of smaller peptides, resulting in lower foam stability and expansion compared to method B.

The presence of emulsified lipids could be another contributing factor to the lower foaming capacity of the extract obtained using method A. Lipids are known to interfere with protein adsorption at the air–water interface, thereby impeding both foam formation and stability [61]. Indeed, the concentration of protein extracted using method A from 1% to 2% further decreased foam stability (Figure 2A). This reduction may also be attributed to the presence of lipids in the extract (3%), which can compete for interfacial space, promote the formation of protein–lipid aggregates, and disrupt the development of cohesive and elastic interfacial films. Albumin, used as a control protein, also showed reduced foam stability at higher concentrations (Figure 2A). This behaviour is consistent with phenomena such as interfacial supersaturation, protein aggregation, and increased rigidity of the interfacial film, all of which can negatively affect foam integrity.

#### 3.3.2. Emulsifying Capacity and Stability

**Figure 3:**
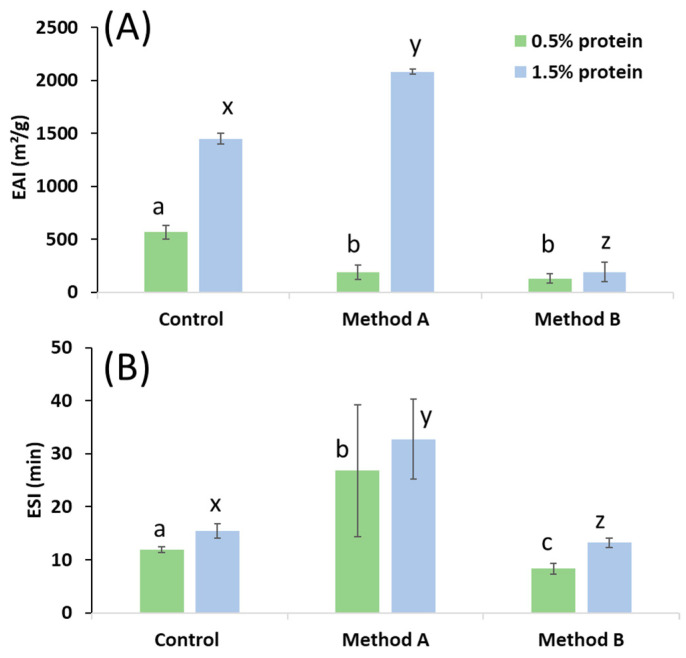
(**A**) Emulsifying activity index (EAI) of protein extracts obtained using methods A or B at two different concentrations (0.5% and 1.5%), compared to commercial albumin (Control); (**B**) emulsifying stability index (ESI) of protein extracts obtained using methods A or B at two different concentrations (0.5% and 1.5%), compared to commercial albumin (Control). Different letters for the same protein concentration (a, b, c or x, y, z) indicate significant differences among samples (*p* < 0.05).

Emulsifying capacity refers to the ability of a protein to stabilise oil-in-water or water-in-oil emulsions. In this study, the emulsifying activity index (EAI) of crab protein was evaluated at two different concentrations (0.5% and 1.5%) to ascertain its ability to stabilise oil-in-water emulsions (Figure 3A).

The results indicated that the EAI of albumin was significantly higher than that of crab protein at a 0.5% concentration (Figure 3A). Notably, the EAI exhibited a significant increase at 1.5% concentration, especially when the protein was derived from method A, achieving the most optimal values compared to protein from method B or albumin. This enhancement can be attributed to the increased number of molecules available at the oil-water interface, resulting in the formation of a thicker and more robust interfacial layer that prevented droplet coalescence.

The ESI of the protein from method A was the highest at concentrations of 0.5 and 1.5% (Figure 3B), whereas the ESI of the protein from method B was significantly lower than that of albumin. The increase in protein concentration led to a slight enhancement in all cases. As observed, the functional properties of the proteins varied depending on the property analysed. This discrepancy could be attributed to the presence of lower molecular weight peptides and hydrophobic amino acid residues, which have been associated with enhanced emulsifying capacity of proteins [60]. These smaller peptides have the capacity to more readily adsorb at the oil-water interface, thereby stabilising the emulsion. The emulsifying capacity of the method A extract may be further enhanced by the residual presence of polar lipids, which is due to the lack of a prior lipid extraction step. In fact, this extract showed a lipid content around 3% (Table 2). These polar lipids may to act as emulsifiers enhancing the ability of the protein extract to stabilise oil-water mixtures.

The contrasting foaming and emulsifying properties of the two extracts emphasise the importance of considering specific functional requirements when selecting an extraction method. While the protein extraction process using method B demonstrated superior foaming properties, method A yielded proteins with superior emulsifying abilities. This discrepancy is likely attributable to the distinct molecular weight profiles and lipid contents of the extracts obtained using the two methods.

### 3.4. Sensory Analysis of a Mayonnaise-Type Sauce

Mayonnaise is a widely appreciated sauce. The primary components of mayonnaise are oil and egg, which, in conjunction, form a semi-solid emulsion characterised by a high fat content (70–80%) along with vinegar or acetic acid. The addition of other ingredients, such as spices (e.g., mustard), is common. The emulsifying properties of eggs, particularly those of their phospholipids and proteins, play a pivotal role in the preparation of this condiment, as they stabilise dispersed oil droplets within the continuous aqueous phase provided by the egg [62]. Nonetheless, substituting egg proteins in mayonnaise offers several potential advantages. Primarily, it can lead to cost reductions for manufacturers, as alternative protein sources may occasionally be more economical than eggs. Another key benefit is the reduction in allergenicity, as replacing eggs can result in sauces suitable for individuals with egg allergies, thereby broadening the consumer base of the product. Consequently, numerous studies have explored the replacement of egg yolk with alternative protein sources, including plant-based options, to ensure the viability of such products for vegan and vegetarian consumers. Examples of egg yolk substitutes include clover sprouts [63] and aquafaba [64], among others [16]. Alternatively, animal-derived proteins, such as caseins [65] or crustacean proteins such as shrimp [20], have been used. From a sustainability perspective, substituting egg protein with plant-based proteins or underutilised bycatch is more environmentally friendly. To achieve successful substitution, the alternative protein source must be carefully selected in conjunction with formulation optimisation to ensure that the desired sensory and functional properties of the sauce are maintained. However, when using seafood protein as a substitute for egg yolk, the potential presence of allergenic proteins, such as tropomyosin, must be considered. This limitation is common to many marine-derived ingredients that have been successfully incorporated into food formulations, and their presence must be indicated on the label to avoid problems among consumers allergic to this protein.

**Figure 4:**
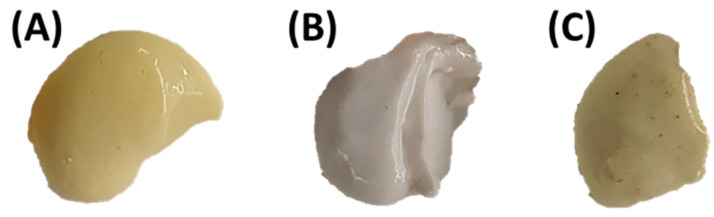
Mayonnaise-type sauces made with egg (control) (**A**), crab protein extract (**B**), and crab protein extract with curry (**C**).

In this study, a mayonnaise-type sauce was formulated by incorporating the protein extract obtained using method A, which exhibited a high emulsifying capacity, as a replacement for the phospholipids in egg yolk. This extract showed a high concentration of protein (90.59 ± 0.34%), and a residual lipid content of 3%. Before preparation of the sauces, the levels of Pb and As in the protein extracts were evaluated. The results showed very low values of Pb (0.12 ± 0.05 ppm) and As (4.33 ± 3.31 ppm), indicating that most of these heavy metals were removed during the protein extraction process. It is important to note that, in a preliminary sensory analysis, the taste of crab protein extract mayonnaise was rated as worse than that of egg mayonnaise, possibly because of the seafood flavour. To improve the taste, a new mayonnaise sauce was prepared with curry in the formulation, as it has an intense flavour, high commercial value in the Asian market, and is becoming increasingly popular in the Western market. Moreover, the incorporation of curry allows for innovation in the range of flavours of these types of sauces. A visual representation of the three sauces is presented in Figure 4.

The mayonnaise prepared with crab protein extract exhibited a whiter coloration, which can be attributed to the presence of carotenoids in the egg yolk, contributing a yellowish hue to the mayonnaise. Conversely, curry, a spice derived from the fruit of the curry tree (*Murraya paniculata*), which is known to contain carotenoids and polyphenols, significantly influences the coloration of products in which it is used as an ingredient.

Regarding the nutritional composition of the sauces (Table 5), the control sample exhibited a lower lipid content (65.5%) than the crab-enriched formulations (67.4–68.3%). In contrast, the sauces containing crab protein showed proportionally higher levels of carbohydrates (1.0–1.4%). About the protein content, it was similar in all the sauces prepared (3.8–4%). The energy density (kcal/g) was slightly higher in the crab formulations (6.9 kcal/g) than in the control (6.5 kcal/g).

**Figure 5:**
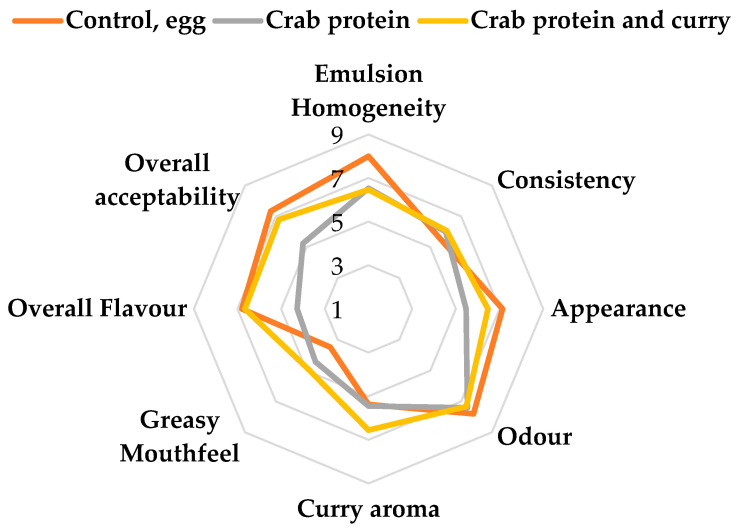
Sensory evaluation scores of the mayonnaise-type sauces represented in a spider graph. The numerical scale ranges from 1 (strongly dislike) to 9 (strongly like).

The sensory evaluation showed that all three sauces were accepted by the panellists. However, clear differences were observed between them (Figure 5).

The control mayonnaise (egg-based) exhibited superior emulsion homogeneity, appearance, overall flavour, and acceptability compared to the formulation containing crab protein. This suggests that the use of crab protein as an ingredient induced a seafood-like taste and a yellowish colour that were perceived as less appealing by the panellists. Similar results were observed by Safira et al. [19] when incorporated a fish roe protein concentrate in a mayonnaise. However, for the attributes of consistency and odour, the scores for both the control and protein-based formulations were comparable, indicating that the seafood odour was not viewed negatively, and the absence of oxidised fat in the protein concentrate [19]. Rajasekaran et al. [20] replaced up to 50% of egg protein with shrimp-derived proteins and noted the development of a fishy aroma, suggesting that mayonnaise formulations may only be acceptable when limited amounts of seafood protein are used. Interestingly, the greasy mouthfeel of crab protein mayonnaise was rated more favourably than that of the control, possibly due to the presence of lipids in the crab protein extract.

The addition of curry to the crab protein formulation effectively mitigated the off-flavours identified by panellists. The use of spices as masking agents has been previously reported by Kirtil [66], and the results of the present study are consistent with this observation. In this study, curry imparted a pleasant and dominant aroma that shaped the overall sensory profile, enabling it to mask or counterbalance the less desirable flavour attributes associated with crab protein. Consequently, the flavour and aroma ratings shifted closer to those of the egg-based control. This improvement in sensory perception was also reflected in the overall acceptability scores, which significantly increased following the inclusion of curry.

## 4. Conclusions

In conclusion, the results highlight the importance of the method used to extract proteins from harbour crabs. Thus, the application of a protein extraction method by precipitation at the isoelectric point, without prior lipid extraction, resulted in a higher protein yield. Furthermore, this protein exhibited better emulsifying properties than commercial albumin. The extract can be incorporated as an ingredient for the preparation of mayonnaise-type sauces, replacing egg yolk, preferably with an ingredient that masks the seafood flavour, such as curry. Allergenicity, mainly related to the presence of tropomyosin, and also to other myofibrillar proteins such as arginine kinase or myosin light chain, can be mitigated through ultraviolet light pulses or other processing strategies. Although such treatments may modify the functional properties of crab proteins, they represent promising approaches for enhancing the safety and applicability of these ingredients. Future research should focus on optimising extraction parameters to maximise foam stability and explore the potential of these proteins in specific product formulations. In addition, the precise molecular weight distribution and composition of protein extracts should be investigated to gain a more complete understanding of the relationship between protein structure and techno-functional properties.

## Figures and Tables

**Table 1:** Formulations used in mayonnaise-type sauces.

	Mayonnaise Sauces		
Ingredient	Control	Crab	Crab + Curry
Egg grade M	1 unit (55 g)	0 g	0 g
Egg white	0 g	34 g	34 g
Crab protein	0 g	2.4 g	2.4 g
Sunflower oil	110 mL	110 mL	110 mL
Sodium chloride	0.7 g	0.7 g	0.7 g
Lemon juice	10 g	10 g	10 g
Curry sauce	0 g	0 g	1 g

**Table 2:** Nutritional composition of the ingredients (in grams) used to prepare the mayonnaise-type sauces. A weight of 51 g was considered for the edible egg components (egg white, 34 g, and egg yolk, 17 g). A density of 0.92 g/mL was considered for the sunflower oil.

	Egg (55 g)	Egg White (34 g)	Curry (1 g)	Crab Protein (2.4 g)	Lemon Juice (10 g)	Sunflower Oil (110 mL)
Lipids	5.1	0.0	0.1	1.6	0.0	101.2
Water	38.7	29.3	0.1	2.8	9.2	0.0
Protein	6.3	3.6	0.1	2.2	0.0	0.0
Carbohydrates	0.5	0.8	0.6	0.0	0.7	0.0
Ash	0.4	0.2	0.1	0.7	0.0	0.0

**Table 3:** Amino acid composition of the whole harbour crab and the protein extracted using methods A or B, expressed as the number of residues per 1000 residues. Asn: Aspartic acid+Asparagine; Glx: Glutamic acid+Glutamine; Hyl: Hydroxylysine; Hyp: Hydroxyproline; EAA: Essential amino acids (*); HAA: Hydrophobic amino acids (^). The total number of essential and hydrophobic amino acids did not include the content in Trp (not determined).

	Analysed Samples		
	Harbour Crab	Proteins (Method A)	Proteins (Method B)
Asx	125 ± 3	121 ± 1	133 ± 4
Threonine *	33 ± 1	48 ± 5	39 ± 1
Serine	56 ± 1	57 ± 2	41 ± 1
Glx	123 ± 1	134 ± 6	173 ± 2
Proline ^	51 ± 2	43 ± 3	37 ± 1
Glycine	142 ± 0	94 ± 0	73 ± 2
Alanine ^	103 ± 1	85 ± 0	87 ± 1
Cysteine	4 ± 0	6 ± 1	11 ± 1
Valine *^	45 ± 2	46 ± 2	59 ± 1
Methionine *^	19 ± 2	30 ± 3	8 ± 0
Isoleucine *^	29 ± 3	36 ± 2	39 ± 0
Leucine *^	61 ± 1	76 ± 2	92 ± 1
Tyrosine	34 ± 1	39 ± 1	35 ± 1
Phenylalanine *^	43 ± 0	56 ± 2	42 ± 1
Hyl	4 ± 0	6 ± 0	0 ± 0
Hyp	3 ± 1	0 ± 0	0 ± 0
Histidine *	13 ± 1	21 ± 1	20 ± 2
Lysine *	63 ± 0	59 ± 0	63 ± 0
Arginine	49 ± 3	43 ± 1	48 ± 2
∑EAA	306 ± 3	372 ± 6	362 ± 1
∑HAA	351 ± 5	372 ± 14	364 ± 3

**Table 4:** Content in macrominerals (Na, K, Ca, Mg, P, S), trace elements (Fe, Cu, Mn, Zn, Cr) and heavy metals (Pb, Cd, As, Hg) of crab. The results were expressed as grams or milligrams per kg of wet weight.

Element	Amount
Sodium	1.23 ± 0.05 (g/kg)
Potassium	0.43 ± 0.02 (g/kg)
Calcium	11.14 ± 1.0 (g/kg)
Magnesium	0.96 ± 0.06 (g/kg)
Phosphorus	1.01 ± 0.03 (g/kg)
Sulphur	0.54 ± 0.02 (g/kg)
Iron	12.32 ± 1.44 (mg/kg)
Copper	2.51 ± 0.18 (mg/kg)
Manganese	1.08 ± 0.05 (mg/kg)
Zinc	4.94 ± 0.41 (mg/kg)
Chrome	0.15 ± 0.02 (mg/kg)
Lead	0.75 ± 0.06 (mg/kg)
Cadmium	0.02 ± 0.00 (mg/kg)
Arsenic	14.86 ± 0.11 (mg/kg)
Mercury	0.06 ± 0.00 (mg/kg)

**Table 5:** Nutritional composition of the three sauces prepared, expressed as %.

Composition	Control	Crab	Crab + Curry
Lipids	65.5	67.4	68.3
Protein	3.9	3.8	4.0
Carbohydrates	0.7	1.0	1.4
Ash	0.3	0.6	0.2
Water	29.5	27.1	26.1
kcal/g	6.5	6.9	6.9

## Data Availability

The original contributions presented in this study are included in the article/Supplementary Material. Further inquiries can be directed to the corresponding author.

## Supplementary Materials

The following supporting information can be downloaded at: https://www.mdpi.com/article/10.3390/foods14234084/s1: Minimal dataset including the raw replicate data used to generate the results presented in the manuscript.
