# Supplementary material for: Valorisation of Protein from Underutilised Harbour Crab (Liocarcinus depurator) as an Egg Yolk Replacer in Mayonnaise-like Sauce Production
Source: Foods. 2025 Nov 28;14(23):4084. doi: 10.3390/foods14234084 (PMC12692170; doi:10.3390/foods14234084)
Supplement: Supplementary file 1 [file foods-14-04084-s001.zip › foods-3959174-supplementary.pdf]

Minimal Dataset Description

The dataset contains the main experimental data supporting the manuscript "Valorisation of Protein from Underutilized Harbour Crab (Liocarcinus depurator) as an Egg Yolk Replacer in Mayonnaise-like Sauce Production." It includes the mineral content, as well as the Z-potential of the extracted protein at different pHs, the results obtained in the sensorial analysis, the nutritional composition of the sauces prepared, as well as the emulsifying and foaming properties of the protein extracted using different methodologies, compared with the properties of albumin at the same concentration.

The dataset is organized in a pdf containing six sheets:

1. Mineral composition of crab protein (Na, K, Ca, Cr, Pb) and the protein fraction (Pb, As), expressed in mg/kg (mean ± SD of at least three replicates).

2. Zeta potential values of crab protein solutions measured at different pH values. Data represent the mean ± SD of three independent measurements.

3. Emulsifying activity index (EAI) and emulsion stability index (ESI) of crab protein at different concentrations and conditions. Values are reported as means ± standard deviations from triplicate measurements.

4. Foaming capacity and stability of crab protein compared with control samples (albumin). Data show the foam height and stability values, expressed as means± standard deviations.

5. Nutritional composition, ingredients and kcalories of the sauces prepared.

6. Sensory evaluation of mayonnaise-like sauces, including parameters such as emulsion homogeneity, consistency, appearance, and odour. Data are expressed as mean.

The dataset is provided as supplementary material and can be accessed alongside the published article. Further details or raw data can be requested from the corresponding author.

Mineral content of Linocarcinus depurator

	mg/Kg				
	**Na**	**K**	**Ca**	**Cr**	**Pb**
	4234.8	1489.5	43776.7	0.46	2.40
	4567.0	1631.8	36447.2	0.57	2.95
	4573.2	1593.0	40790.2	0.61	2.75
Average	4458.3	1571.4	40338.0	0.55	2.70
desvest	193.61	73.56	3685.62	0.07	0.28
	**Fe**	**Cu**	**Mn**	**Zn**	**Mg**
	38.64	8.33	3.75	16.20	3673.6
	46.79	9.42	3.86	18.76	3270.4
	48.37	9.48	4.11	18.74	3452.8
Average	44.60	9.08	3.90	17.90	3465.6
desvest	5.22	0.65	0.18	1.47	201.9
	**Cd**	**P**	**S**	**As**	**Hg**
	0.05	3513.7	1891.9	53.51	0.22
	0.06	3721.3	2015.0	54.07	0.22
	0.06	3718.5	1943.4		0.21
	0.06	3651.2	1950.1	53.79	0.22
Average	0.06	3697.0	1969.5	53.93	0.22
desvest	0.00	39.71	39.55	0.20	0.01

As and Pb content of protein from Method A

	mg/kg	
	**As**	**Pb**
	6.35	0.16
	2.34	0.10
	7.87	0.17
	0.77	0.07
Average	4.33	0.13
desvest	3.33	0.05

Z-Potential at different pH values

Type	Sample Name	Temperature (	Zeta Potential (mV)
Zeta	CRAB pH 2.5 dilution 1/100 1	25.1	39.6
Zeta	CRAB pH 2.5 dilution 1/100 2	25	42.7
Zeta	CRAB pH 2.5 dilution 1/100 3	24.9	45.1
Zeta	CRAB pH 3 dilution 1/100 1	24.9	31.7
Zeta	CRAB pH 3 dilution 1/100 2	25	28.6
Zeta	CRAB pH 3 dilution 1/100 3	25.1	29.1
Zeta	CRAB pH 3.5 dilution 1/100 1	24.9	21.1
Zeta	CRAB pH 3.5 dilution 1/100 2	25.1	23.3
Zeta	CRAB pH 3.5 dilution 1/100 3	25	23.5
Zeta	CRAB pH 4 dilution 1/100 1	25	15.6
Zeta	CRAB pH 4 dilution 1/100 2	24.9	15.2
Zeta	CRAB pH 4 dilution 1/100 3	25	14.6
Zeta	CRAB pH 4.5 dilution 1/100 1	25	4.17
Zeta	CRAB pH 4.5 dilution 1/100 2	25	4.59
Zeta	CRAB pH 4.5 dilution 1/100 3	25	4.6
Zeta	CRAB pH 4.65 dilution 1/100 1	24.9	0.675
Zeta	CRAB pH 4.65 dilution 1/100 2	25	0.456
Zeta	CRAB pH 4.65 dilution 1/100 3	25	0.811
Zeta	CRAB pH 4.65 dilution 1/100 1	24.9	0.408
Zeta	CRAB pH 4.65 dilution 1/100 2	25	0.571
Zeta	CRAB pH 4.65 dilution 1/100 3	25	0.471
Zeta	CRAB pH 4.77 dilution 1/100 1	25.1	-2.21
Zeta	CRAB pH 4.77 dilution 1/100 2	25	-2.05
Zeta	CRAB pH 4.77 dilution 1/100 3	24.9	-2.32
Zeta	CRAB pH 5 dilution 1/100 1	24.9	-5.84
Zeta	CRAB pH 5 dilution 1/100 2	25.1	-6.08
Zeta	CRAB pH 5 dilution 1/100 3	25	-6.16
Zeta	CRAB pH 6 dilution 1/100 1	25	-24
Zeta	CRAB pH 6 dilution 1/100 2	24.9	-30.9
Zeta	CRAB pH 6 dilution 1/100 3	25	-27.4
Zeta	CRAB pH 7 dilution 1/100 1	24.9	-30.7
Zeta	CRAB pH 7 dilution 1/100 2	25.1	-35.9
Zeta	CRAB pH 7 dilution 1/100 3	25	-35.2
Zeta	CRAB pH 8 dilution 1/100 1	24.9	-41.9
Zeta	CRAB pH 8 dilution 1/100 2	25.1	-35.9
Zeta	CRAB pH 8 dilution 1/100 3	25	-40.4
Zeta	CRAB pH 9.2 dilution 1/100 1	25.1	-29.6
Zeta	CRAB pH 9.2 dilution 1/100 2	25	-36.7
Zeta	CRAB pH 9.2 dilution 1/100 3	24.9	-34.7
Zeta	CRAB pH 10.1 dilution 1/100 1	25.1	-38.7
Zeta	CRAB pH 10.1 dilution 1/100 2	25	-38.3
Zeta	CRAB pH 10.1 dilution 1/100 3	24.9	-41.8
Zeta	CRAB pH 11 dilution 1/100 1	25.1	-38.1
Zeta	CRAB pH 11 dilution 1/100 2	25	-41.9
Zeta	CRAB pH 11 dilution 1/100 3	24.9	-39.8
Zeta	CRAB pH 12 dilution 1/100 1	25	-31.7
Zeta	CRAB pH 12 dilution 1/100 2	24.9	-34.9
Zeta	CRAB pH 12 dilution 1/100 3	25	-35.2
Zeta	CRAB pH 12.2 dilution 1/100 1	24.9	-23
Zeta	CRAB pH 12.2 dilution 1/100 2	25.1	-22.3

pH	Average	Std deviation
2.5	42.47	2.76
3	29.80	1.66
3.5	22.63	1.33
4	15.13	0.50
4.5	4.45	0.25
4.65	0.57	0.15
4.77	-2.19	0.14
5	-6.03	0.17
6	-27.43	3.45
7	-33.93	2.82
8	-39.40	3.12
9.2	-33.67	1.92
10.1	-39.60	0.25
11	-39.93	1.90
12	-33.93	2.33
12.2	-22.65	0.49

Emulsifying activity, Average for each sample

AVERAGE

crab 0,5%	Control	A	B
EAI (m^2/g)	567	189	130
ESI (min)	11.9	26.8	8.3

DESV

crab 0,5%	Control	A	B
EAI (m^2/g)	66	66	45
ESI (min)	0.6	12.4	1.1

AVERAGE

crab 1,5%	Control	A	B
EAI (m^2/g)	1448	2081	191
ESI (min)	15.4	32.7	13.2

DESV

crab 1,5%	Control	A	B
EAI (m^2/g)	49	25	93
ESI (min)	1.4	7.5	0.9

Foaming stability along time

	Time (min)	CRAB 1% Average control	Average A	Average B
	Vi(mL)	10	10.00	10.00
Height (cm)	0	15	11.00	1.67
	1	14	10.00	1.67
	5	12	9.00	1.63
	10	12	9.00	1.60
	20	11	8.00	1.43
	30	10	8.00	1.23
	60	10	7.00	1.00

	Time (min)	CRAB 2% Average control	Average A	Average B
	Vi(mL)	10	10.00	10.00
Height (cm)	0	22.25	16.00	23.67
	1	21.5	10.67	23.67
	5	17.25	8.67	23.33
	10	16	8.00	23.00
	20	15.5	7.00	21.33
	30	14.5	7.00	19.33
	60	14.25	7.00	17.00

FOAMING STABILITY (FS) AND FOAMING EXPANSION (FE)

	CRAB 1%			
	FE (%)	FS(%)	FE (%)	FS(%)
	AVERAGE		STD DEVIATION	
Control	50.0	67.0	14.1	6.3
Method A	15.0	64.0	7.1	5.8
Method B	136.7	71.4	20.8	17.0

	CRAB 2%		Std deviation	
	FE (%)	FS(%)	FE (%)	FS(%)
	AVERAGE		STD DEVIATION	
Control	131.7	60.0	3.5	2.6
A	60.0	43.9	10.0	2.7
B	173.3	86.6	5.8	4.4

COMPOSITION AND CALORIES OF THE SAUCES PREPARED

	Mayonnaise sauces		
Ingredient	Control	Crab	Crab + Curry
Egg grade M**	51 g	0 g	0 g
Egg white	0 g	34 g	34 g
Crab protein	0 g	2.4 g	2.4 g
Sunflower oil*	110 mL (101.2 g)	110 mL (101.2 g)	110 mL (101.2 g)
Sodium chloride	0.7 g	0.7 g	0.7 g
Lemmon juice	10 g	10 g	10 g
Curry sauce	0 g	0 g	1 g
total (g)	162.9	148.3	149.3

* Considered a density of 0.92 g/mL

** A total weight of 55 g was considered, 51 g of which were egg white and egg yolk

Composition/100 g, in grams	Egg	Egg white	Curry	Crab protein	Lemon juice	Sunflower oil
Lipids	10.0	0.0	14.0	3.0	0.2	100
Water	75.8	86.3	8.8	5.1	92.3	0
Protein	12.4	10.7	14.3	90.6	0.4	0
Carbohydrates	1.0	2.4	55.8	0.0	6.9	0
Ash	0.9	0.7	7.1	1.3	0.2	0
TOTAL	100	100	100	100	100	100

Composition of each ingredient (g)	Egg (55 g)	Egg white (34 g)	Curry (1 g)	Crab protein (2.4g)	Lemon juice (10 g)	Sunflower oil (110 ml)
Lipids	5.1	0.0	0.1	1.6	0.0	101.2
Water	38.7	29.3	0.1	2.8	9.2	0.0
Protein	6.3	3.6	0.1	2.2	0.0	0.0
Carbohydrates	0.5	0.8	0.6	0.0	0.7	0.0
Ash	0.4	0.2	0.1	0.7	0.0	0.0

Calories of each ingredient	Egg (55 g)	Egg white (34 g)	Curry (1 g)	Crab protein (2.4g)	Lemon juice (10 g)	Sunflower oil (110 ml)
Lipids	45.7	0.0	0.0	0.0	0.2	910.8
Water	0.0	0.0	0.0	0.0	0.0	0.0
Protein	25.3	14.6	0.6	8.7	0.1	0.0
Carbohydrates	2.0	3.2	2.2	0.0	2.8	0.0
Ash	0.0	0.0	0.0	1.5	0.0	0.0
TOTAL	73.0	17.8	2.8	10.2	3.1	910.8

	Mayonnaise sauces		
Calories for each ingredient	Control	Crab	Crab + Curry
Egg grade M	73	0	0
Egg white	0	18	18
Crab protein	0	10	10
Sunflower oil	990	990	990
Sodium chloride	0	0	0
Lemmon juice	3	3	3
Curry sauce	0	0	3
TOTAL	1066	1021	1024
kcal/g	6.5	6.9	6.9

Composition (g)	Control	Crab	Crab + Curry
Lipids	106.3	102.9	101.4
Protein	6.4	5.8	6.0
Carbohydrates	1.2	1.5	2.1
Ash	0.5	1.0	0.3
Water	47.9	41.4	38.7
TOTAL	162.2	152.6	148.4

Composition (%)	Control	Crab	Crab + Curry
Lipids	65.5	67.4	68.3
Protein	3.9	3.8	4.0
Carbohydrates	0.7	1.0	1.4
Ash	0.3	0.6	0.2
Water	29.5	27.1	26.1

Sensorial analysis. Individual scores for each panellist

Control	1	2	3	4	5	6	7	8	9	10	11	12	AVERAGE
Emulsion Homogeneity	8.2	8.2	8.2	8.2	7.4	8.2	8.2	8.2	8.2	8.2	8.2	6.6	8.00
Consistency	6.6	5	6.6	4.2	5	8.2	5	6.6	4.2	5.8	5.8	4.2	5.60
Appearance	6.6	6.6	4.2	8.2	7.4	8.2	7.4	7.4	7.4	7.4	8.2	6.6	7.13
Odour	7.4	6.6	8.2	7.4	8.2	8.2	7.4	7.4	8.2	8.2	8.2	8.2	7.80
Curry aroma	6.8	3.7	3.7	5.0	5.9	6.8	6.8	5.0	5.9	5.0	5.0	5.0	5.38
Greasy Mouthfeel	4.4	2.1	7.9	3.3	3.3	2.1	2.1	3.3	4.4	1.0	2.1	5.6	3.48
Overall Flavour	6.6	5	6.6	6.6	7.4	6.6	5.8	7.4	8.2	8.2	6.6	6.6	6.80
Overall acceptability	7.4	7.4	7.4	7.4	7.4	6.6	7.4	7.4	8.2	8.2	7.4	5.8	7.33
**Crab**	**1**	**2**	**3**	**4**	**5**	**6**	**7**	**8**	**9**	**10**	**11**	**12**	
Emulsion Homogeneity	6.6	6.6	3.4	7.4	6.6	6.6	7.4	3.4	8.2	8.2	7.4	6.6	6.53
Consistency	6.6	6.6	6.6	5.8	5.8	3.4	5	5.8	8.2	6.6	5.8	5.8	6.00
Appearance	6.6	6.6	5.8	4.2	5.8	4.2	8.2	3.4	2.6	5.8	8.2	4.2	5.47
Odour	6.6	7.4	8.2	6.6	8.2	5	7.4	6.6	8.2	8.2	8.2	8.2	7.40
Curry aroma	5.9	6.8	5.0	5.0	5.0	5.0	6.8	5.0	5.9	5.0	5.0	5.0	5.45
Greasy Mouthfeel	4.4	4.4	7.9	4.4	3.3	3.3	1.0	5.6	4.4	7.9	2.1	4.4	4.43
Overall Flavour	4.2	6.6	5.8	6.6	5.8	2.6	5	2.6	1	6.6	1	3.4	4.27
Overall acceptability	5.8	8.2	5.8	5	6.6	3.4	5.8	2.6	1	7.4	1.8	2.6	4.67
**Crab and curry**	**1**	**2**	**3**	**4**	**5**	**6**	**7**	**8**	**9**	**10**	**11**	**12**	
Emulsion Homogeneity	5	8.2	4.2	4.2	5.8	8.2	8.2	8.2	4.2	8.2	7.4	5.8	6.47
Consistency	6.6	8.2	6.6	5.8	4.2	8.2	5	5.8	4.2	6.6	5.8	5.8	6.07
Appearance	5	8.2	4.2	6.6	5	8.2	7.4	6.6	5	6.6	8.2	6.6	6.47
Odour	6.6	6.6	7.4	5.8	5.8	8.2	7.4	7.4	8.2	8.2	8.2	8.2	7.33
Curry aroma	6.3	9.0	7.2	4.6	9.0	7.2	8.1	6.3	5.4	3.7	5.4	6.3	6.56
Greasy Mouthfeel	4.4	6.7	7.9	4.4	3.3	4.4	3.3	5.6	4.4	9.0	2.1	3.3	4.90
Overall Flavour	3.4	4.2	8.2	8.2	8.2	8.2	8.2	8.2	7.4	5.8	5.8	4.2	6.67
Overall acceptability	3.4	6.6	8.2	8.2	8.2	8.2	7.4	7.4	8.2	5.8	5	5	6.80
